# Activation of miR-21 by STAT3 Induces Proliferation and Suppresses Apoptosis in Nasopharyngeal Carcinoma by Targeting PTEN Gene

**DOI:** 10.1371/journal.pone.0109929

**Published:** 2014-11-03

**Authors:** Hesheng Ou, Yumei Li, Min Kang

**Affiliations:** 1 College of Pharmacy, Guangxi Medical University, Nanning City, Guangxi Province, P.R. China; 2 The First Affiliated Hospital, Guangxi Medical University, Nanning City, Guangxi Province, P.R. China; H. Lee Moffitt Cancer Center & Research Institute, United States of America

## Abstract

The present study is to investigate the role of microRNA-21 (miR-21) in nasopharyngeal carcinoma (NPC) and the mechanisms of regulation of PTEN by miR-21. Fifty-four tissue samples were collected from 42 patients with NPC and 12 healthy controls. Human NPC cell lines CNE-1, CNE-2, TWO3 and C666-1 were used for cell assays. To investigate the expression of miR-21, RT-PCR was employed. RT-PCR, Western blotting, and immunohistochemistry were used to measure the expression of STAT3 mRNA and STAT3 protein. To test the effect of miR-21 on the cell growth and apoptosis of NPC cells *in vitro*, transfection of CNE1 and CNE2 cell lines and flow cytometry were performed. TUNEL assay was used to detect DNA fragmentation. To validate whether miR-21 directly recognizes the 3′-UTRs of PTEN mRNA, luciferase reporter assay was employed. miR-21 expression was increased in NPC tissues compared with control and the same result was found in NPC cell lines. Notably, increased expression of miR-21 was directly related to advanced clinical stage and lymph node metastasis. STAT3, a transcription factor activated by IL-6, directly activated miR-21 in transformed NPC cell lines. Furthermore, miR-21 markedly inhibited PTEN tumor suppressor, leading to increased AKT activity. Both *in vitro* and *in vivo* assays revealed that miR-21 enhanced NPC cell proliferation and suppressed apoptosis. miR-21, activated by STAT3, induced proliferation and suppressed apoptosis in NPC by targeting PTEN-AKT pathway.

## Introduction

Nasopharyngeal carcinoma (NPC) causes 80,000 new cases and 50,000 deaths every year [1], [2]. NPC is mainly a non-lymphomatous, non-keratinizing, squamous cell carcinoma, which is highly malignant with abilities of local invasion and early distant metastasis [1] Genetic susceptibility, endemic environment factors, and Epstein-Barr virus infection are believed to be the major etiologic factors of NPC [3]–[5]. Currently, the standard care for these patients consists of concurrent chemoradiotherapy with cisplatin-based regimens, generally followed by adjuvant chemotherapy. The 5-year survival rate for patients with NPC remains about 70%. However, systemic and local side effects caused by chemotherapy greatly tormented the patients physically and psychologically. Therefore, it is of importance to study the precise molecular mechanisms of NPC and explore new, safe and effective NPC therapies.

MicroRNAs (miRNAs) are small non-coding RNAs (20 to 24 nucleotides) that post-transcriptionally modulate gene expression by negatively regulating the stability or translational efficiency of their target mRNAs [6]. Increasing data showed that miRNAs played an important role in cancer, and a concept of “oncomirs” was proposed [7]. Among them, miR-21 emerged as a key oncomir, since it was consistently up-regulated in a wide range of cancers [8]–[11], and implicated in multiple malignancy-related processes such as cell proliferation, apoptosis, invasion, and metastasis [12]–[15]. Functional studies showed that knockdown of miR-21 led to reduced proliferation and tumor growth in MCF-7 cells [16], [17], and reduced invasion and metastasis in MDA-MB-231 cells [17]. These data clearly implicated that miR-21 acted as a key molecule in carcinogenesis. However, the mechanisms by which miR-21 acts in the development of NPC still remain unknown, and no miR-21 targets were reported in NPC.

Persistent activation of signal transducer and activator of transcription (STAT) has been observed and is frequently associated with malignant transformation [18]. Constitutive activation of STAT proteins, notably of STAT3, is detected in many human tumor cells and cells transformed by oncoproteins [19]–[21]. STAT3 is a well-characterized transcription factor that has been demonstrated to contribute to various processes of tumorigenesis, such as tumor cell survival and proliferation, invasion, angiogenesis and drug resistance [22]. Aberrant STAT3 enhances uncontrolled growth and survival of cancer cells through dysregulation of gene expression, including cyclin D1 [23], c-Myc [24], and survivin genes [25], and hence, contributing to tumorigenesis.

The enzyme phosphatase and tensin homologue (PTEN) gene is one of the most frequently inactivated tumor suppressor genes in a variety of cancers. Inactivating mutations and deletions of the PTEN gene are found in many types of cancers, including NPC [26]. It is reported that PTEN gene inhibits Akt activation (phosphorylation) [27]–[28], which plays a central role in an outermost complex network of cell growth modulation that affects protein biosynthesis, cell cycle arrest and apoptosis [29], [30]. Interestingly, 3′-UTR of PTEN gene has been proved to harbor a putative binding site for miR-21 by bioinformatics tools. Therefore, we hypothesize that PTEN gene is regulated by miR-21 as one of the several miR-21 target genes in NPC. The present study is to investigate the role of miR-21 in NPC and the mechanisms of regulation of PTEN by miR-21.

## Materials and Methods

### Patients and tissue samples

Fifty-four tissue samples were collected from 42 patients with NPC and 12 healthy controls. The 42 NPC patients comprised 20 early cases and 22 advanced cases, whose clinical and pathological data were displayed in Table 1. Tissue samples were immediately frozen in liquid nitrogen after resection and stored at −80°C until use. Both tumor and non-tumor samples were confirmed by the pathological examinations. The clinical stage was defined according to the 2002 AJCC/UICC staging classifications. The pathological stage, grade, and nodal status were appraised by an experienced pathologist. Clinicopathologic characteristics including gender, age, pathology, differentiation, and tumor-node-metastasis staging have been collected.

**Table 1:** Clinicopathologic characteristics of NPC patients aged between 30 and 74, with a median age of 48.

Characteristics		Number of patients (%)
Gender	Male	31/42 (73.8)
	Female	11/42 (26.2)
Age (years)	<60	30/42 (71.4)
	≥60	12/42 (28.6)
TNM stage	I+II	20/42 (47.6)
	III+IV	22/42 (52.4)
Lymph node metastasis	Positive	24/42 (57.1)
	Negative	18/42 (42.9)
Distant metastasis	Positive	5/42 (11.9)
	Negative	37/42 (88.1)
Pathologic type	WHO II	4/42 (9.5)
	WHO III	38/42 (90.5)
Smoking and alcohol consumption history	Smoking	30/42 (71.4)
	Non-smoking	12/42 (28.6)
	Alcoholic	26/42 (61.9)
	Non-alcoholic	16/42 (38.1)

Note: Primary tumors at pharynx nasalis in NPC had deep, wide and irregular invasions into peripheral tissues, so the sizes of these tumors could not be measured in the evaluation of NPC staging. None of the NPC patients received any treatment before. Before standard chemotherapies or radiotherapies, tissue biopsies from pharynx nasalis were examined. For subsequent treatment for NPC, concurrent chemoradiotherapy plus adjuvant chemotherapy were performed.

Written informed consent was obtained from all participants. Collections and using of tissue samples were approved by the The Human Research Ethics Committee of The First Affiliated Hospital of Guangxi Medical University. The procedures are in accordance with the Helsinki Declaration of 1975.

### Cell culture, transfection and establishment of stable cell lines

Human NPC cell lines CNE-1, CNE-2, TWO3 and C666-1 were purchased from Cell Application (Santiago, USA) and were cultured in Dulbecco's Modified Eagle Medium containing 10% fetal bovine serum, 100 IU/ml penicillin and 100 µg/ml streptomycin in humidified 5% CO_2_ atmosphere at 37°C as previously described [31]. Primary cells between passage 4 and 10 were used in the experiments and in the establishment of stable cell lines. For transfection, cells were grown up to 90% confluency, and were transfected with small interfering RNA (siRNA) or miRNA plasmids using Lipofectamine 2000 (Life Technologies, USA) by incubating with OptiMem-I media for 4 h. The cells were then transferred into fresh Dulbecco's Modified Eagle Medium with 10% fetal bovine serum. To establish stable cell lines with miRNA overexpression, G418 (1 mg/ml; Invitrogen, USA) was added into the medium 2 days after transfection with selective medium being changed once a week. The transfected cells expressing neomycin-resistant gene survived in the selective medium with the elimination of the non-transfected cells. The cells were individually picked 2 weeks later using conventional cloning techniques for expansion. Passages 2–10 of the stable cell lines were identified by verifying miRNA expression with Northern blotting and *in situ* hybridization as previously described [32], and were used for experiments.

### Immunohistochemistry

The selected tumor tissues were used to construct tissue microarray slides. Paraffin sections were cut and mounted on glass slides, and 5 µm sections from formalin-fixed and paraffin-embedded specimens were deparaffinized using xylene and rehydrated in graded ethanol. Samples were then preincubated with 3% H_2_O_2_ to eliminate endogenous peroxidase activity. Antigen retrieval was achieved by heating the sections for 2 min at 100°C in citric acid buffer (0.01 mol/L, pH 6.0). Immunohistochemistry was performed by the two-step method using primary antibody, including heat-induced antigen-retrieval procedures. Sections were incubated overnight at 37°C with primary antibody. After the primary antibody was washed off, the components of the EnVision Detection System were applied with an anti-mouse polymer (EnVision1/HRP/Mo, Dako, Glostrup, Denmark). The primary antibodies used were all mouse anti-human monoclonal antibodies against STAT3, BCL-2 and BAX (1∶100 dilution; American Research Products, Belmont, USA). Negative controls were treated identically but with the primary antibody being omitted.

Immunoreactivity was evaluated independently by three researchers. The percentage of positive tumor cells was determined by each observer, and the average of the 3 scores was calculated. Ten high-power fields were randomly selected and 1,000 cells were counted in each core. When the mean of percentage of positive cells was close to 0% or 100%, the standard deviation (SD) was close to 0. When the mean was approximately 50%, the SD was approximately 5%. Therefore, the SD did not increase with the mean. The following categories were used for scoring: none (0), mild (1), moderate (2), and strong (3) for the intensity of staining; <5% (0), 5–25% (1), 25–50% (2), and>50% (3) for the percentage of positive staining. The combination of the intensity and percentage of staining resulted in the following scores: 0–1, negative (-); 2–4, moderate (+); 5–6 strong (++) [33], [34].

### Real-time polymerase chain reaction (RT-PCR) analysis

Total RNA was extracted using Trizol reagent (Invitrogen, USA) for miR-21, STAT3 mRNA and PTEN mRNA analyses. For the detection of miR-21 expression, stem-loop RT-PCR was performed as previously described [35]. Quantitative PCR (qPCR) was carried out using SYBR Premix Ex Taq (Takara, Japan) according to the manufacturer's protocol. Relative expression was evaluated by comparative CT method and normalized to the expression of U6 small RNA. The primers for miR-21 are as follows: stem-loop RT primer, 5′-GTCGTATCCAGTGCAGGGTCCGAGGTATTCGCACTGGATACGACTCAACA-3′; forward primer, 5′-GCCCGCTAGCTTATCAGACTGATG-3′, and reverse primer, 5′-GTGCAGGGTCCGAGGT-3′. The primers for U6 are as follows: RT primer, 5′-GTCGTATCCAGTGCAGGGTCCGAGGTATTCGCACTGGATACGACAAAAATATG-3′; forward primer, 5′-GCGCGTCGTGAAGCGTTC-3′, and reverse primer, 5′-GTGCAGGGTCCGAGGT-3′. For the detection of STAT3 mRNA and PTEN mRNA expression, qPCR was performed using QuantiTect SYBR Green PCR Kit (Qiagen, Germany). Glyceraldehyde-3-phosphate dehydrogenase (GAPDH) was used to normalize STAT3 mRNA and PTEN mRNA expression levels. The forward and reverse primer sequences for STAT3 mRNA were 5′-CTGTCAGATGCCAAATGC-3′ and 5′-CTTACCGCTGATGTCCTT-3′, respectively. The forward and reverse primer sequences for PTEN mRNA were 5′-GAGGGATAAAACACCATG-3′ and 5′-AGGGGTAGGATGTGAACCAGTA-3′, respectively. The forward and reverse primer sequences for GAPDH were 5′-AACTTTGGCATTGTGGAAGG-3′ and 5′-ACACATTGGGGGTAGGAACA-3′, respectively. All the experiments were performed in triplicate.

### Western blotting analysis

Cells were washed, harvested, and prepared for Western blotting as previously described [36]. A total of 30 µg protein was separated using 8% sodium dodecyl sulfate polyacrylamide gel electrophoresis and transferred onto a nitrocellulose membrane. The membrane was incubated with the primary antibodies for STAT3, AKT and PTEN (1∶1000), and then actin (1∶5000) in 5% non-fat milk in Tris-buffered saline and Tween 20 at room temperature for 1 h, followed by exposure to a goat anti-rabbit or anti-mouse secondary antibody conjugated with horseradish peroxidase. Signals of the immunoreactive bands were visualized using the electrochemiluminescent detection system.

### Transfection of anti-miR-21 into NPC cells

CNE1 and CNE2 cells was maintained in Dulbecco's Modified Eagle Medium, supplemented with 100 U/ml penicillin, 100 µg/ml streptomycin and 10% fetal bovine serum (Invitrogen, USA). For transfection, the anti-miR-21 or control, and miR-21 high expression plasmid or control (Exiqon A/S, Denmark) were delivered at a final concentration of 50 nM using Lipofectamine 2000 reagent (Life Technologies, USA).

### Cell proliferation and colony formation assay

Cells were plated in 96-well plates (5000 cells/well), incubated for 48 h, and then transfected with 50 nM miR-21 mimics, its inhibitor or negative control. At the end of the incubation, cell proliferation reagent WST-8 (10 µl) was added to each well and incubated for 3 h at 37°C. Viable cell numbers were estimated by measuring optical density at 450 nm.

For the colony formation assay, 50 nM miR-21 mimics or its inhibitor was transfected into CNE1 cells, which were cultured in medium containing 10% fetal bovine serum, with the medium being refreshed every 3 days. After incubation for 14 days, cells were fixed with methanol and stained with 0.1% crystal violet (Sigma-Aldrich, USA). Visible colonies were manually counted. Triplicate wells were measured in each group.

### Annexin V-fluorescein isothiocyanate (FITC)/propidium iodide (PI) double staining

The Annexin-V-FITC/PI double staining assay was used to detect cellular apoptosis. CNE1 and CNE2 cells were transfected with 50 nmol/l of miR-21 mimics or negative control. At the end of incubation, the cells were collected, washed with cold PBS and resuspended at 1×10^6^ cells/ml in Annexin-V binding buffer. The supernatant (100 µl/tube) was incubated with 5 µl Annexin-V-FITC and 5 µl PI for 15 min at room temperature in the dark. Binding buffer (400 µl) was then added to each tube, followed by cytometric analysis within 1 h of staining. All experiments were repeated for three times.

### Animals

Five- to six-week-old female BALB/c-nude mice (Shanghai Laboratory Animal Co., Ltd., China) were used for experimental tumorigenicity assays. To facilitate estrogen-dependent xenograft establishment, each mouse received 17-estradiol (20 mg/kg; Sigma-Aldrich, USA) intraperitoneally once a week. One week after the treatment, equivalent amount of CNE1 cells, treated with miR-21 or control (100 nM for 48 h; Panagene, Inc., Korea) without transfection reagents according to the manufacturer's protocol, were injected subcutaneously (10^7^ cells/tumor) into the right axilla of nude mice [37]. The mice were weighed, and tumor width (W) and length (L) were measured every day. Tumor volume was estimated according to the standard formula V = π/6×L×W^2^, as described previously [38]. Animals were killed 9 days after the initial growth of the CNE1 xenografts was detectable, and the tumors were extracted. In all experiments, the ethics guidelines for investigations in conscious animals were followed, with approval from the Ethics Committee for Animal Research at The First Affiliated Hospital of Guangxi Medical University.

### Terminal deoxynucleotidyl transferase dUTP nick end labeling (TUNEL)

Nude mice were euthanized and frozen sections were prepared as described above. TUNEL assay was performed according to the manufacturer's instructions. Sections were visualized under a fluorescent microscope (Nikon, USA).

### Luciferase reporter assay

The 3′-UTR segments of PTEN mRNA containing miR-21 binding sites were amplified by PCR from human genomic DNA, inserted into the Xba1-site of pGL3 vector (Promega, USA), and named as pGL3-PTEN-wt. Using pGL3-PTEN-wt as a template, mutations were introduced into the predicted miR-21 binding sites by QuikChange site-directed mutagenesis kit (Stratagene, USA), and named as pGL3-PTEN-mut. CNE1 cells were transfected in 24-well plates with wild-type or mutant reporter plasmid vector by Lipofectamine 2000, and the cells were transfected again with miR-21 inhibitor or negative control at 6 h after the second transfection. After 36 h, luciferase activity was measured using dual luciferase assay system (Promega, USA). The firefly luciferase activity of each sample was normalized to Renilla luciferase activity.

### Statistical analyses

Spearman's rank correlation test was used for correlation analysis between predicted target gene protein levels and endogenous miR-21 levels measured previously by RT-PCR [39]. Pearson's Chi-Square tests were used to compare target gene expression levels to clinicopathological characteristics. Survival curves were estimated by the Kaplan-Meier method and log-rank test. All analyses were performed using SPSS 16.0 for Windows (SPSS Inc., USA). All tests were two-tailed, and P<0.05 was considered statistically significant.

## Results

### miR-21 is overexpressed in tissues and cell lines from NPC patients

**Figure 1:**
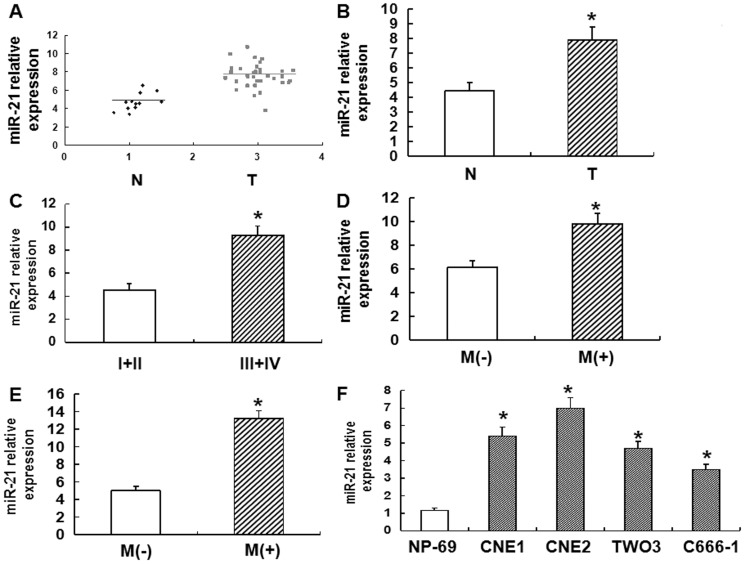
Expression of miR-21 in various tissues or cell lines. **(A)** Relative expression of miR-21 in non-tumor tissues (n = 12) and NPC tissues (n = 42). RT-PCR was used to determine the expression of miR-21, which was normalized to U6 expression to obtain relative expression. N, non-tumor tissues; T, tumor tissues. **(B)** Histograms of the average relative expression of miR-21 in non-tumor tissues (n = 12) and NPC tissues (n = 42). Data are means ± SD. Asterisks indicate values that are significantly different from the values of non-tumor tissues (P<0.05). **(C)** Histograms of the average relative expression of miR-21 in patients at stages I+II and III+IV. Data are means ± SD. Asterisks indicate values that are significantly different from the values of patients at stage I+II (P<0.05). **(D)** Histograms of the average relative expression of miR-21 in patients without and with distant metastasis. Data are means ± SD. Asterisks indicate values that are significantly different from the values of patients without distant metastasis (P<0.05). M, distant metastasis. **(E)** Histograms of the average relative expression of miR-21 in patients without and with lymph node metastasis. Data are means ± SD. Asterisks indicate values that are significantly different from the values of patients without lymph node metastasis (P<0.05). M, lymph node metastasis. **(F)** Histograms of the average relative expression of miR-21 in normal nasopharyngeal epithelial cell line (NP-69) and NPC cell lines (CNE1, CNE2, TWO3, and C666-1). Data are means ± SD. Asterisks indicate values that are significantly different from the values of NP-69 (P<0.05).

To investigate the expression of miR-21, RT-PCR was employed. Our data showed that the expression of miR-21 in tissues from NPC patients was significantly higher than that in tissues from normal person (P<0.01) (Fig. 1A and B). As the advance of clinical tumor-node-metastasis stage (Fig. 1C), distant metastasis (Fig. 1D), and lymph node metastasis (Fig. 1E), the expression of miR-21 was enhanced. In addition, the expression of miR-21 in NPC cell lines CNE1, CNE2, TWO3, and C666-1 was elevated compared with that in normal nasopharyngeal epithelial cell line NP-69 (P<0.01) (Fig. 1F). These data indicated that miR-21 was overexpressed in tissues and cell lines from NPC patients.

### STAT3 is up-regulated in NPC

**Figure 2:**
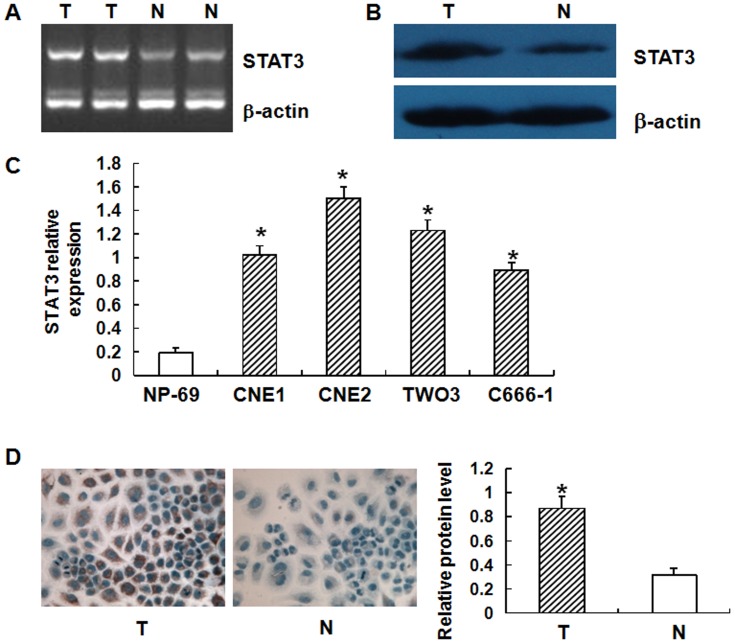
STAT3 expression in various tissues and cell lines. **(A)** Relative STAT3 mRNA levels in NPC tissues (T) and non-tumor tissues (N) determined by RT-PCR. The results were normalized to β-actin mRNA levels and were presented as relative STAT3 mRNA expression. Asterisks indicate values that are significantly different from the values of non-tumor tissues (P<0.01). **(B)** Relative expression of STAT3 protein in NPC tissues (T) and non-tumor tissues (N) determined by Western blotting. STAT3 protein expression was normalized to β-actin protein expression. Asterisks indicate values that are significantly different from the values of non-tumor tissues (P<0.01). **(C)** Relative expression of STAT3 mRNA in four NPC cell lines (CNE1, CNE2, TWO3, and C666-1) and normal nasopharyngeal epithelial cell line (NP-69) determined by RT-PCR. Asterisks indicate values that are significantly different from the values of NP-69 (P<0.01). **(D)** Immunohistochemical staining and quantification of expression of STAT3 in NPC cell line CNE1 and normal nasopharyngeal epithelial cell line NP-69. The cytoplasm was counter-stained with hematoxylin. N, NP-69; T, CNE1.

To measure the expression of STAT3 mRNA and STAT3 protein, RT-PCR, Western blotting, and immunohistochemistry were used. The results showed that the levels of STAT3 mRNA and protein were significantly increased in NPC tissues compared with normal samples (Fig. 2A and B). In addition, the level of STAT3 mRNA was also enhanced in CNE1, CNE2, TWO3, and C666-1 cells compared with NP-69 cells (Fig. 2C). Immunohistochemical data also showed that STAT3 protein levels in CNE1 cells were higher than that in NP-69 cells. These data suggested that STAT3 was up-regulated in NPC.

### STAT3 regulates the activity of miR-21 during transformation

**Figure 3:**
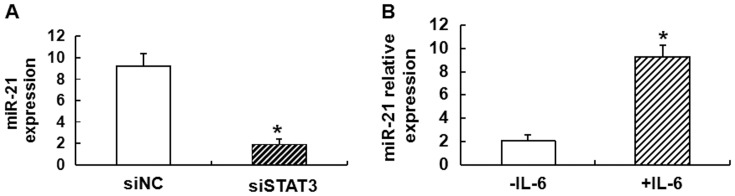
Expression of miR-21 regulated by STAT3 during transformation. **(A)** Levels of miR-21 expression in CNE1 cells after treatment with siSTAT3 and siNC. siSTAT3 indicates that STAT3 is inhibited by siRNA, while siNC indicates that STAT3 is not inhibited by siRNA. Data are means ± SD. Asterisks indicate values that are significantly different from the values of siNC group. **(B)** Levels of miR-21 expression without (-IL-6) and with (+IL-6) IL-6 treatment of CNE1 cells. -IL-6 indicates that STAT3 is not activated by IL-6, while +IL-6 indicates that STAT3 is activated by IL-6. Data are means ± SD. Asterisks indicate values that are significantly different from the group without IL-6 treatment.

To study how STAT3 regulates the activity of miR-21 during transformation, STAT3 binding to the targets sites in the miR-21 promoter was induced by interleukin (IL)-6 upon transformation, and the expression of miR-21 was measured by RT-PCR. The results showed that inhibition of STAT3 by siRNA strongly reduced miR-21 expression levels (Fig. 3A). By contrast, STAT3 activation by IL-6 in CNE1 cells resulted in up-regulation of miR-21 (Fig. 3B). These results strongly suggested that STAT3 function in transformation involved transcriptional activation of miR-21.

### miR-21 stimulates NPC cell growth and inhibits apoptosis *in vitro*

**Figure 4:**
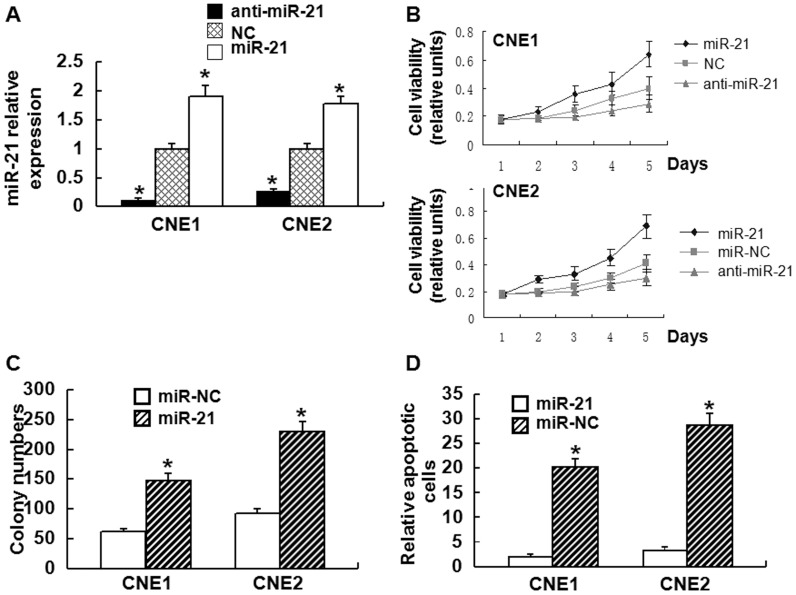
The effect of miR-21 on NPC cell growth and apoptosis *in vitro*. **(A)** Relative expression of miR-21 in CNE1 and CNE2 cells stably transfected by empty vector (NC) or vectors expressing miR-21 and anti-miR-21 detected by RT-PCR. The results were normalized to U6 expression and expressed as fold change relative to the corresponding negative control. anti-miR-21 is the inhibitor of miR-21. Data are means ± SD. Asterisks indicate values that are significantly different from the NC group (n = 3). **(B)** Proliferation curve of CNE1 and CNE2 cells stably transfected by empty vector (NC) or vectors expressing miR-21 and anti-miR-21. Data are means ± SD. Asterisks indicate values that are significantly different from the NC group (n = 3). **(C)** Representative results of colony formation of CNE1 and CNE2 cells transfected with miR-21 mimics and miR control (NC). Data are presented as means ± SD. Asterisks indicate values that are significantly different from the NC group. **(D)** The apoptosis of CNE1 and CNE2 cells transfected with empty vector (NC) or vector expressing miR-21 detected by flow cytometry. Data are means ± SD. Asterisks indicate values that are significantly different from the NC group.

To test the effect of miR-21 on the cell growth and apoptosis of NPC cells *in vitro*, transfection of CNE1 and CNE2 cell lines and flow cytometry were performed. After transfection, the miR-21 levels was increased by 90% in CNE1 cells, and 78% in CNE2 cells (P<0.01), while knockdown of miR-21 by anti-miRNA reduced miR-21 levels by 88% in CNE1 cells, and 73% in CNE2 cells (P<0.01) (Fig. 4A). miR-21 led to an increase in CNE1 and CNE2 cell growth and proliferation (P<0.05, Fig. 4B and C). By contrast, anti-miR-21 decreased CNE1 and CNE2 cell growth (P<0.01) (Fig. 4B). Flow cytometry data showed that overexpression of miR-21 significantly inhibited CNE1 and CNE2 apoptosis compared with the control (P<0.05) (Fig. 4D). These data demonstrate miR-21 stimulated NPC cell growth and inhibited apoptosis *in vitro*.

### miR-21 promotes tumor growth and suppresses apoptosis *in vivo*

**Figure 5:**
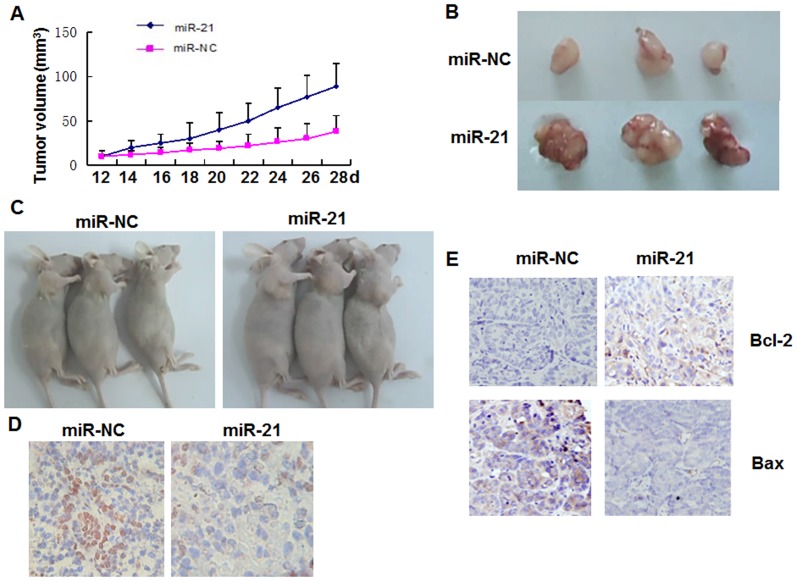
*In vivo* effect of miR-21 on CNE1 cell growth in nude mice. CNE1 cells (10^7^ cells/tumor), treated with miR-21 or control (100 nM for 48 h) without transfection reagents, were injected subcutaneously into the right axilla of nude mice. **(A)** Growth curves for CNE1 cells treated with miR-21 (n = 8) and control (n = 8) obtained by *in vivo* proliferation assay. P<0.05 for all. **(B)** Photographs of tumors from miR-21 and control groups. **(C)** Photographs of mice from miR-21 and control groups. **(D)** Representative photomicrographs of CNE1 cells and apoptosis in miR-21 and control groups detected by TUNEL assay. **(E)** Representative photomicrographs of immunohistochemical staining and the relative levels of Bcl-2 and Bax on xenograft tumor sections obtained from mice in miR-21 and control groups. The cytoplasm was counter-stained with hematoxylin (×400).

To investigate the *in vivo* effect of miR-21, equal numbers (3×10^7^) of CNE1 cells treated with miR-21 or the control were subcutaneously injected into nude mice (eight mice per treatment), and TUNEL and immunohistochemistry assays were performed. Detectable tumor masses (0.011±0.014 g) were observed in all mice in the control group, while much larger tumors (0.056±0.032 g) were seen in all mice in the miR-21 group (P<0.05) (Fig. 5B and C). Data of tumor volume showed that CNE1 cells treated with miR-21 led to larger tumors more rapidly (Fig. 5A) than CNE1 control cells in nude mice. In addition, TUNEL assay data showed that fewer cells were undergoing DNA fragmentation after miR-21 treatment in mice (Fig. 5D). Cell survival in the early phase of apoptotic cascade depends mostly on the balance between pro-apoptotic and anti-apoptotic proteins of the Bcl-2 family. Bcl-2 family consists of anti-apoptotic factors such as Bcl-2 and pro-apoptotic factors such as Bax. In this study, we tested whether Bcl-2 and Bax protein levels were affected by miR-21. Furthermore, the expression of BCL-2 protein was significantly up-regulated, and the expression of BAX protein was dramatically down-regulated in the miR-21 treatment group compared with the control group (Fig. 5E). These results demonstrated that miR-21 promoted tumor growth and suppressed apoptosis *in vivo*.

### miR-21 targets the PTEN tumor suppressor gene that functions through the Akt pathway

**Figure 6:**
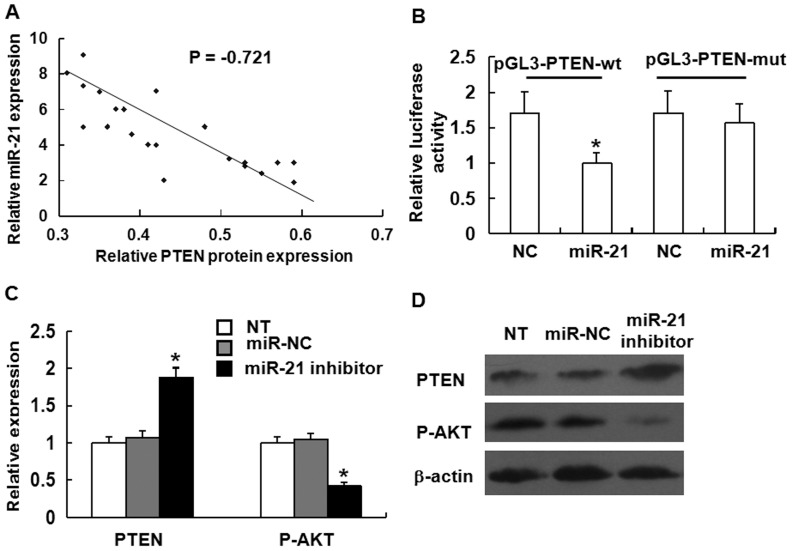
miR-21 regulates PTEN expression at the post-transcriptional level and influences the phosphorylation of AKT. Cells were transfected with miR-21 inhibitor, inhibitor-negative control (NC) or blank control culture medium (NT). Cell lysates were obtained after 48h for analysis. **(A)** Correlation between miR-21 expression and PTEN protein levels in NPC tissues (Pearson correlation = −0.721). **(B)** Relative luciferase activity of wt or mutant reporter plasmid co-transfected into CNE1 cells with miR-21 or negative control (NC). Luciferase activity was normalized to that of the control group to obtain relative luciferase activity. pGL3-PTEN-wt represents pGL3-PTEN wild-type plasmid vector, and pGL3-PTEN-mut represents pGL3-PTEN mutant reporter plasmid vector. Data were means ± SD (n = 3). Asterisks indicate values that are significantly different from the NC group (P<0.01). **(C)** Expression of PTEN and phosphorylated AKT examined by Western blotting. The results were normalized to β-actin protein expression and expressed as fold change relative to the corresponding negative control. Data were means ± SD (n = 3) Asterisks indicate values that are significantly different from the NT group (P<0.01). **(D)** Representative immunoblots of PTEN protein expression and the levels of phosphorylated AKT after treatment with negative control and miR-21 inhibitor.

To measure the expression levels of miR-21 and PTEN mRNA in tissues and to validate whether miR-21 directly recognizes the 3′-UTRs of PTEN mRNA, we cloned a sequence with the predicted target sites of miR-21 or a mutated sequence with the predicted target sites to downstream of the pGL3 luciferase reporter gene and employed Western blotting assay. Statistical analysis showed that significant inverse correlation was observed between the levels of miR-21 and PTEN protein (Fig. 6A). In addition, inhibition of miR-21 expression results in up-regulation of PTEN mRNA expression (Fig. 6C and D). Similarly, inhibition of miR-21 expression decreased the levels of phosphorylated AKT (Fig. 6C and D). When wild-type (wt) or mutant vector was transfected with miR-21, the luciferase activity of wt vector was significantly decreased (P<0.001) compared with mutant vector (Fig. 6B). By contrast, there was no significant difference between wt and mutant vector when transfected with negative control miRNA. These data suggested that miR-21 regulated PTEN expression at the post-transcriptional level and influenced the phosphorylation of AKT.

## Discussion

In this study, we showed that miR-21 and STAT3 were elevated in the tissues and cell lines of NPC. Up-regualtion of STAT3 is responsible for the activation of miR-21 during cellular transformation. In addition, we demonstrated that miR-21 promoted proliferation and suppressed apoptosis in NPC cell lines *in vitro* and *in vivo*. Finally, we showed that PTEN expression, as well as Akt phosphorylation, was regulated by miR-21 in NPC cell lines.

miR-21 was recently recognized as one of the most important biomarkers implicated in human malignancy. The up-regulation of miR-21 was observed in diverse types of malignancies such as lung cancer [10], [40]–[43], breast cancer [11], [44], gastrointestinal cancer [45]–[46], hepatocellular cance [12], [47], prostate cancer [48]–[49], pancreatic cance [50] bladder cancer [51], esophageal cancer [14], [52], NK-cell lymphoma [53], laryngeal carcinoma [54], and tongue squamous cell carcinomas [55]. In some types of cancers, high levels of miR-21 expression were linked to poor prognosis [10], [55]–[58]. However, it was not clear whether miR-21 expression was changed in NPC. In the present study, we showed that miR-21 expression was significantly higher in NPC tissues and cell lines. Further, the up-regulation of miR-21 was observed in 29 out of 42 NPC tissues compared with non-tumor tissues. Specifically, 14 out of 22 NPC patients at stage III–IV showed miR-21 overexpression, while 15 out of 20 NPC patients with low miR-21 expression presented stage I-II disease. This study also showed miR-21 overexpression in 18 out of 24 NPC patients with lymph node metastasis, and 4 out of 5 patients with distant metastasis. Therefore, up-regulation of miR-21 occurred in NPC, and was associated with the stage of the disease. In CNE1, CNE2, TWO3, and C666-1 cells, the up-regulation of miR-21 was further confirmed by RT-PCR to play an important role in cell transformation. Thus, miR-21 was up-regulated in NPC, and associated with the clinical stage, as well as cancer metastasis.

The molecular mechanisms involved in the miR-21 regulation in cancer still remain unclear. Recent data showed that some transcription factors play an important role in miRNA expression. For example, p53/p63/p73 family binding sites modulate promoter activity of miRNAs of the miR-200 family, which are known regulators of cancer stem cells and epithelial-mesenchymal transitions [59]. STAT3-mediated overexpression of miR-17 blocked BIM protein expression and caused resistance to AZD6244 [60]. Activation of IL-6-STAT3 signaling caused the up-regulation of miR-23a expression in hepatocellular carcinoma [61]. These data suggested that transcription factors and miRNAs are associated with human cancers. In fact, in non-cancer cells, we previously described an intronic miRNA that suppresses the proliferation of vascular endothelial cells via the inhibition of STAT3 signaling [62]. In this study, we provided strong evidence that the activation of miR-21 induced by STAT3 is important for cellular transformation. We found that STAT3 directly binds three sites in miR-21 promoter regions and is required for transcriptional induction of these miRNAs. On the other hand, inhibition of STAT3 by siRNA strongly reduced miR-21 expression levels. Conversely, STAT3 activation by IL-6 treatment in CNE1 cells resulted in the up-regulation of miR-21, suggesting that STAT3 and miR-21 are required for cellular transformation. This study further confirmed that STAT3 acts as a key molecule in the regulation of miRNA in cellular events and human diseases.

PTEN gene was first discovered and identified as a tumor suppressor gene [63], [64]. It was well known that PTEN induces apoptosis [65], [66] and controls cell growth [67] and angiogenesis [68] through interfering several signaling pathways. The key target of PTEN is phosphatidylinositol 3, 4, 5-trisphosphate [69]. PTEN is one of the most frequently inactivated tumor suppressors in different tumor types. Several mechanisms such as genetic mutation, promoter methylation, and post-transcriptional modification, may contribute to PTEN inactivation. In this study, it was found that PTEN expression was significantly down-regulated in NPC. PTEN expression was significantly increased, while the levels of phosphorylated Akt were decreased by miR-21 inhibitor. These findings suggest that the main mechanism of the reduction of PTEN protein is miRNAs regulation at post-transcriptional level. Recently, PTEN was reported as a direct target of miR-21 that was involved in miR-21-mediated effects on tumor biology: cell growth, migration, and invasion in human hepatocellular carcinoma [12] as well as gemcitabine-induced apoptosis in human cholangio carcinoma [69]. In this study, we observed a significant negative correlation between miR-21 and PTEN protein in NPC. We postulated that miR-21 played an important role in the regulation of cell growth and apoptosis in NPC.

This postulation was verified by our experimental studies on NPC cell lines. miR-21 expression was increased in CNE1 and CNE2 cell lines, and cell growth was significantly increased in these cells transfected with miR-21. Meanwhile, cell apoptosis was significantly decreased by transfection of these cells with miR-21. These alterations concur with the observation in cancer tissues that the pro- and anti-apoptotic genes were changed. As discussed above, PTEN-AKT pathway was associated with miR-21 expression. It is reasonable to speculate that miR-21 promotes cell growth and/or inhibits apoptosis that is linked to PTEN expression. Given that PTEN inhibits tumor cell growth and invasion by blocking the PI3K/AKT pathway [70], we reached a conclusion that miR-21 induces proliferation and inhibits apoptosis in NPC by targeting PTEN-AKT pathway.

In summary, miR-21 is overexpressed in NPC tissues, and promotes cell growth and suppresses apoptosis in NPC cell lines. These effects are linked to direct suppression of PTEN by miR-21. Our data suggested that miR-21 is a potential therapeutic target for NPC.
